# Contribution of a positive psychology-based conceptual framework in reducing physician burnout and improving well-being: a systematic review

**DOI:** 10.1186/s12909-021-03021-y

**Published:** 2021-11-25

**Authors:** Shahrzad Bazargan-Hejazi, Anaheed Shirazi, Andrew Wang, Nathan A. Shlobin, Krystal Karunungan, Joshua Shulman, Robert Marzio, Gul Ebrahim, William Shay, Stuart Slavin

**Affiliations:** 1 College of Medicine, Charles R. Drew University of Medicine and Science, 1731 E. 120th St, Los Angeles, CA 90059 USA; 2 David Geffen School of Medicine, University of California, Los Angeles, CA USA; 3 Department of Psychiatry, School of Medicine, University of California San Diego, San Diego, CA USA; 4 Feinberg School of Medicine, Northwestern University, Chicago, IL USA; 5 Accreditation Council for Graduate Medical Education, Chicago, USA

**Keywords:** Well-being; burnout, Physician, Positive emotion, Engagement, Meaning, Relationship, Achievement

## Abstract

**Background:**

The PERMA Model, as a positive psychology conceptual framework, has increased our understanding of the role of Positive emotion, Engagement, Relationships, Meaning, and Achievements in enhancing human potentials, performance and wellbeing. We aimed to assess the utility of PERMA as a multidimensional model of positive psychology in reducing physician burnout and improving their well-being.

**Methods:**

Eligible studies include peer-reviewed English language studies of randomized control trials and non-randomized design. Attending physicians, residents, and fellows of any specialty in the primary, secondary, or intensive care setting comprised the study population. Eligible studies also involved positive psychology interventions designed to enhance physician well-being or reduce physician burnout. Using free text and the medical subject headings we searched CINAHL, Ovid PsychINFO, MEDLINE, and Google Scholar (GS) electronic bibliographic databases from 2000 until March 2020. We use keywords for a combination of three general or block of terms (Health Personnel OR Health Professionals OR Physician OR Internship and Residency OR Medical Staff Or Fellow) AND (Burnout) AND (Positive Psychology OR PERMA OR Wellbeing Intervention OR Well-being Model OR Wellbeing Theory).

**Results:**

Our search retrieved 1886 results (1804 through CINAHL, Ovid PsychINFO, MEDLINE, and 82 through GS) before duplicates were removed and 1723 after duplicates were removed. The final review included 21 studies. Studies represented eight countries, with the majority conducted in Spain (*n* = 3), followed by the US (*n* = 8), and Australia (n = 3). Except for one study that used a bio-psychosocial approach to guide the intervention, none of the other interventions in this review were based on a conceptual model, including PERMA. However, retrospectively, ten studies used strategies that resonate with the PERMA components.

**Conclusion:**

Consideration of the utility of PERMA as a multidimensional model of positive psychology to guide interventions to reduce burnout and enhance well-being among physicians is missing in the literature. Nevertheless, the majority of the studies reported some level of positive outcome regarding reducing burnout or improving well-being by using a physician or a system-directed intervention. Albeit, we found more favorable outcomes in the system-directed intervention. Future studies are needed to evaluate if PERMA as a framework can be used to guide system-directed interventions in reducing physician burnout and improving their well-being.

**Supplementary Information:**

The online version contains supplementary material available at 10.1186/s12909-021-03021-y.

## Background

Physician mental health burnout is a public health problem in the United States [1–10]. Physician burnout is associated with negative consequences, such as physician-reported error [11] medication error, [12, 13] suicide, [1, 14] substance abuse, [15] sick leave, [16] physician turnover, [17] decreases in best practice, reduction in physician empathy, lower patient satisfaction, reduced health outcome, and increase cost [18] among the others. The system-related drivers of physician burnout include meaningless excessive workload, [19] work-home conflicts, [20] hours worked at nights on call, [21] and a negative work environment. [22] Individual related factors such as personality, interpersonal skills, and coping behaviors are also responsible. [23] It is evident that addressing physician burnout may benefit from a multi-dimensional approach, in which both physicians and the system are responsible for developing thoughtful solutions that consider the drivers of burnout. [10, 24, 25].

The advocates of positive psychology (PI) have voiced the need for a new paradigm to approach burnout. [26, 27] A ground-breaking approach, positive psychology utilizes optimum human potentials, strengths, and functioning to allow individuals to thrive. [28, 29] The positive psychology approach specifically focuses on the constructs of feeling good and functioning well, including hedonic and eudaimonic wellbeing. [30–33] Positive psychology interventions target mechanisms of feelings, thoughts, and behavior via strategies such as gratefulness, savoring, mindfulness, acts of kindness, forgiveness, meaningful activities to achieve positive health and wellbeing. [34–37] Similarly, Seligman has conceptualized psychological wellbeing within the Positive emotion (focusing on optimistic perspectives in endeavors and relationships), Engagement (participation in enjoyable activates that stretches the intellect, skills, and emotional capacities), Relationships (fostering meaningful social connections), Meaning (utilization of logic, religion, and spirituality to find the impact of endeavors to self and society that leads to purposeful living), and Accomplishments (accompaniment of goals and recognition to develop a sense of fulfillment) (PERMA). This model of wellbeing provides a framework to promote understanding of the elements that can be targeted to maximize life satisfaction and creativity. [29, 38] According to Seligman, the PERMA model, provides a framework based on which individuals can realize their core strengths and uniqueness to strive to achieve optimal functioning. [28, 29, 32] Studies examining PERMA domains reveal improvements in life satisfaction and creativity, protection against stress, [39] augmented wellbeing, [40] and reduction in depressive symptoms, [37] as well as decreased job burnout.[41] Others have reported a positive association between PERMA’s components and productivity and happiness at work. [42] Additionally, the number of published studies applying PERMA to improve the elements of feeling good and functioning well among nurses and college students has rapidly increased. [31, 43].

The existing literature on allied health professionals indicates the PERMA framework may offer a useful foundation to develop proactive interventions to address physician well-being. However, the value of PERMA as a conceptual framework to reduce physician burnout or improve physician well-being has not been systematically evaluated. To address this gap, we conducted a systematic review to characterize the contribution and outcome of the PERMA conceptual framework in interventional studies aiming to improve physician well-being. Our findings may benefit in the aim of developing interventions targeted to reduce burnout and improve well-being among physicians.

## Methods

### Data source and literature searches

We reported a systematic review according to the Preferred Reporting Items for Systematic Reviews and Meta-analyses (PRISMA) guidelines. We developed our search strategy with an expert librarian (JS). He ran two separate searches in Ovid PsychINFO, separating terms within each group with an “OR.” He combined those two searches in a third search where both group of concepts were combined with “AND.” Conceptually the search was the same as the PubMed search, but we reported the results in the Supplementary Table 1 using Ovid’s syntax for a combination of two general or block of terms (Health Personnel OR Health Professionals OR Physician OR Internship and Residency OR Medical staff Or Fellow) AND (Burnout) AND (Positive Psychology OR PERMA OR Wellbeing Intervention OR Well-being Model OR Wellbeing Theory). Using free text and controlled vocabulary we searched CINAHL, Ovid PsychINFO, and MEDLINE, from 2000 until March 2020. We searched the CINAHL with Full-Text database using the EBSCOhost Research Platform. We used the Ovid research platform to search the PsychINFO database. For searches conducted in Ovid PsycINFO, we identified index terms in the Ovid thesaurus and included narrower terms to create a more comprehensive search. Filters were applied to all searches, limiting the date range from 2000 to 2020. We did not apply the English language filter during the search stage. To improve the comprehensiveness of the search, we supplemented the search by adding Google Scholar (GS).[44] We used a combination of the aforementioned key terms. For example, we queried for: Positive Psychology OR PERMA OR Wellbeing Intervention OR Well-being Model OR Wellbeing Theory. The GS search engine uses stemming technology, which morphologically correlates similar words to match documents with different forms of the same word [45], as a result our research yielded approximately 16,000 items (February 2020). [46] We used a stop policy by limiting our search to the first 300 hits sorted by relevance, to better manage the massive results. Additionally, we searched the references of the eligible studies and included relevant studies as well as relevant systematic reviews found from screening reference lists of eligible studies. A review protocol was registered a priori through PROSPERO (CRD205059). No funding was received.

### Eligibility criteria

Eligible studies include peer-reviewed English language studies of randomized control trials and non-randomized design. Attending physicians, residents, and fellows of any specialty in the primary, secondary, or intensive care setting comprised the study population. Eligible studies, also, involved positive psychology interventions designed to enhance physician well-being or reduce physician burnout.

### Exclusion criteria

Interventional studies not focusing on physician well-being or burnout from positive psychology perspectives were excluded. We also excluded studies that focused on medical students, as well those not published in peer-review journals, including gray literature.

### Study selection

**Fig. 1:**
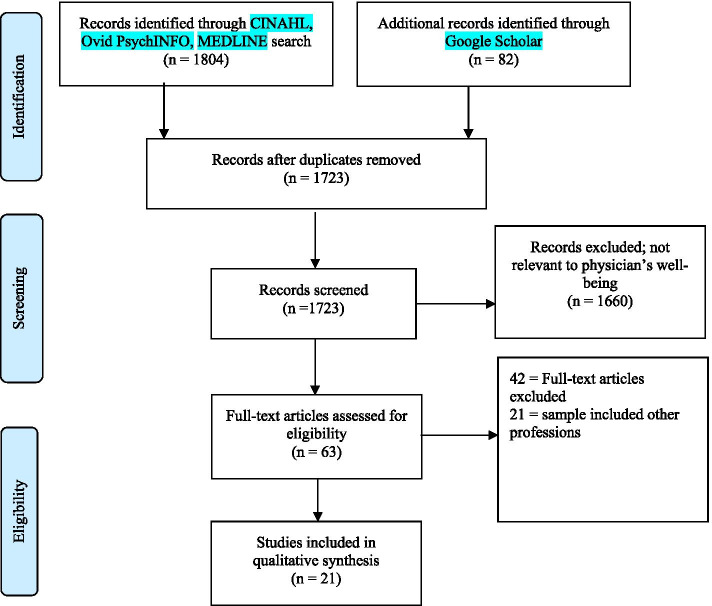
PRISMA [89] Flow chart of study selection to evaluate interventional frameworks for physician well-being [89].

Once we merged research results in a citation manger software, Endnote (X9; Clarivate, Philadelphia, PA, USA) (Fig. 1), we removed duplicates. The remaining articles were shared by three members of the research team (KD, AS, RM) via an online shared folder. The screening process was conducted in two steps. First, members of the research team independently screened the titles and abstracts and selected relevant studies. We considered studies relevant at this stage if they included positive psychology, enhancing wellbeing or wellness, mitigating/reducing burnout terms in their title, or physician wellness or wellbeing in the study abstract or keywords. Second, the selected abstracts were reviewed and validated with another reviewer with content expertise (SB). The full texts of the potentially relevant reports were retrieved and screened by all study team members for final inclusion based on inclusion and exclusion criteria. To do this, the Liberian (JS) exported all articles from the database to the EndNote reference management system. He created three copies of the EndNote references and distributed them to the three reviewers. Each reviewer worked on her/his copy of the EndNote file. In the first reviewing round, the reviewers independently read titles and abstracts to decide whether a reference is potentially relevant to the study. Each reviewer created an ‘*Includes’* and ‘*Excludes’* folder in their EndNote library and placed the respective references from the general reference list into either of these folders. In the next step, the reviewers compared included references with each other. After consensus was reached, the full texts of the included titles and abstracts were reviewed by each reviewer independently, working on their copies of the EndNote library. After reading all articles, each reference in the library was discussed in detail to gain consensus.

During all screening steps, disagreements among the reviewers were discussed and reconciled (k = 0.73, substantial agreement).

### Data extraction and synthesis

Reviewers (AW, NS, KK) independently abstracted data and completed risk of bias assessments from the included studies into a Microsoft Excel Spreadsheet (V2016; Microsoft, Redmond, WA, USA) and validated with another reviewer with content expertise (SB). Improved physician well-being articles were summarized according to the authors, the country where the study was conducted, study design, number of participants, participant clinical title (attending or resident), medical specialty, risk of bias (low-high), and the quality grade (A-E). The risk of bias for each included study was determined by adapting designations from the RoB 2 tool for the risk of bias in study designs. [47] The quality grade was determined by adapting grades detailed by [48] that are shown in Supplementary Table 2.

Included studies were also summarized according to the study aim, intervention, intervention level (physician or organization level), intervention domain, wellbeing conceptualization domain (physical, mental, work, social) application of PERMA, and the beneficiary of study intervention (intervention, control, none, both). Any disagreements in data abstraction, risk of bias assessments, and synthesis were resolved through discussion (k = 0.88, almost perfect agreement).

### Analytical plan

Our analytical approach focused on the contribution of the PERMA model and its components as the underlying theoretical framework to inform the selected study interventions. Study characteristics were summarized as counts, range, and proportions. Study aims and interventional components were interpreted and descriptively summarized. Scoring agreements were expressed as a weighted Cohen’s Kappa coefficient (k = < 0.20 as slight, 0.21–0.40 as fair, 0.41–0.60 as moderate, 0.61–0.80 as substantial, and > 0.81 as almost perfect agreement). When appropriate Stata/IC (v16.1; StataCorp LP, College Station, TX, USA) statistical software was used for all quantitative analyses.

## Results

Our search retrieved 1886 results (1804 through CINAHL, Ovid PsychINFO, MEDLINE, and 82 through GS) before duplicates were removed and 1723 after duplicates were removed. Of the 1723 references, 1660 were excluded. Ultimately 63 full-text articles were evaluated, and 21 studies met final inclusion (Fig. 1). The full-text studies exclusively targeted reducing physician burnout, stress or enhancing physician well-being by using positive psychology interventions (Table 1). Studies represented eight countries, with the majority conducted in the US (*n* = 8), followed by Spain (*n* = 3), and Australia (n = 3). Of the 21 net studies, 18 were randomized control trials, and three studies were non-randomized. The number of participants in each study ranged in size: 1–50 (n = 8), 51–100 (*n* = 5), 100+ (n = 8). While most participants were attendings (*n* = 13), seven studies delineated the medical specialty of the attendings: primary care (*n* = 6), internal medicine (*n* = 4), and pediatrics (*n* = 3). Overall, studies had low-risk bias (*n* = 17) and were of grade A scientific quality (n = 17).

**Table 1:** Characteristics of Included Studies Aimed at Improving Physician Well-being

Authors	`Country	Study Design	Number of Participants	Study Participants	Medical Specialty	Risk of Bias	Quality Grade
Ali et al., [50]	USA	Cluster RCT	45	Attending	Critical care	Low	A
Amutio et al., [56]	Spain	RCT & Quasi-experimental	42	Physician	Primary care	Low	A
Asuero et al., [57]	Spain	RCT	68	Health care professional including physicians	Primary care	Low	A
Axisa, et al., [62]	Australia	RCT	46	Resident	Pediatrics	Low	A
Bragard, et al., [68]	Belgium	RCT	96	Resident	Oncology	Low	A
Dyrbye, et al., [67]		RCT	290	Attending	Multiple	Low	A
Garland, et al., [49]	Canada	RCT	24	Attending	Critical care	Low	A
Gunasingam, et al., [61]	Australia	RCT	31	Resident	–	Low	A
Holt & Del Mar [88]	Australia	RCT	233	Attending	Primary care	Low	A
Linzer, et al., [52]	USA	Cluster RCT	166	Attending	Primary care	Low	A
Lucas, et al., [53]	USA	Cluster RCT	62	Attending	Internal medicine	Low	A
Margalit, et al., [63]	Israel	RCT	102	Attending	Primary care	Low	A
Martins, et al., [65]	Argentina	RCT	74	Resident	Pediatrics	Low	A
Milstein, et al., [66]	USA	RCT	15	Attending	Pediatrics	Low	A
Montero-Marin, et al., [58]	Spain	Pre-Post quasi-experimental	290	Attending	Primary care	Low	A
Parshuram, et al., [55]	Canada	Cluster RCT	49	Resident	Multiple	Moderate	C
Ripp, et al., [51]	USA	Retrospective cohort	239	Resident	Internal medicine	Moderate	B
Shea, et al., [54]	USA	RCT	106	Resident	Internal medicine	Low	A
Verweij, et al., [59]	Netherlands	Controlled before-&-after	50	Attending	Primary care	Moderate	B
Weight, et al., [64]	USA	Controlled before-&-after	628	Resident, fellow	Multiple	Moderate	B
West, et al., [60]	USA	RCT	74	Attending	Multiple	Low	A

[50, 56, 57, 62, 68, 67, 49, 61, 88, 52, 53, 63, 65, 66, 58, 55, 51, 54, 59, 64, 60]

As reflected in Table 2, the majority of the interventions were physician-directed (n = 13) and eight targeted the system within which physicians practiced, and only one study intervened with both the physician and the system. System-directed interventions targeted work hour schedule, staffing, and workload to reduce burnout. [49]^,^[50]^,^[51]^,^[51–55] Of the physician-directed interventions five studies utilized mindfulness exercises, [56–60] six utilized some types of group activities such as debriefing sessions, [61] group discussion, [57, 62, 63] and team-based. [64] Eight studies involved some sort of individualized practice in reducing burnout or to enhance well-being including exercise, [57, 64] role-play, [63] self-care activities, [65–67], and communication skill training. [52, 68] Of ten studies demonstrating favorable outcome (statistically significant findings benefiting the intervention group), six were system-directed intervention, and four were physician-direct intervention. Eleven studies reported no statistically significant results, of which three implemented system-directed intervention and eight implemented physician-directed intervention. One study incorporated both system and physician directed intervention and reported positive outcomes. [60] Except Margalit, et al., [63] who used a bio-psychosocial approach to guide their intervention, none of the other interventions in this review were based on a conceptual model, including PERMA. However, retrospectively,10 studies used strategies that resonate with the PERMA components. For example seven studies used mindfulness strategies to improve positive emotion (*n* = 7), [56–60] and four studies used physician-related or system-related strategies to enhance participant meaningfulness in work, engagement in work, and professional supporting relationship (*n* = 4) [59–61, 67] to reduce burnout or enhance well-being.

**Table 2:** Interventions of Included Studies Aimed at Improving Physician Well-being

Study	Aim	Intervention	Intervention Level	Use of Conceptual Model including PERMA	Study Outcome
Ali et al., [50]	To reduce job distress and enhance home-life balance	Receiving weekend breaks and lower workload	System-directed	No	Favorable Outcome for the Intervention
Amutio et al., [56]	To alleviate work stress-related symptoms	Participation in a two-phase mindfulness-based stress reduction program with maintenance; an 8-week mindfulness-based stress reduction-based psycho-educational program followed by10 additional months in reducing physicians’ stress	Physician-directed	No	Favorable outcome for the intervention group
Asuero et al., [57]	To reduce burnout and mood disturbance, increase empathy, and develop mindfulness.	Participation in 8 weekly workshops of 2.5 h each over 2 months on coping strategies, mindfulness practices, yoga and group discussion plus one additional 8-h retreat session.	Physician-directed	No	Favorable outcome for the intervention group
Axisa, et al., [62]	To promote wellbeing	Participation in a half day workshop/group work activity to encourage discussion about approaches to work, life and self-care	Physician-directed	No	No effect
Bragard, et al., [68]	To manage stress in interviews, stress to communicate in interviews, and burnout.	Participation in 30-h training workshops to enhance communication skills and a 10-h stress management skills training in small groups (seven or less)	Physician-directed	No	No effect
Dyrbye, et al., [67]	To enhance well-being (including engagement and meaning in work)	Completing one weekly self-directed micro-tasks from a menu of 5–6 tasks over a 10 weeks period	Physician- directed	No	No effect
Garland, et al., [49]	To reduce burnout	Having an intensivist around-the-clock (24 h) in intensive care units. Taking night calls from home as opposed to staying in ICU.	System-directed	No	Favorable outcome for the intervention group
Gunasingam, et al., [61]	To reduce levels of burnout	Receiving four confidential debriefing sessions every two weeks for approximately 1 h after the working day, onsite at the hospital by experienced senior health professionals. Discussion topics emerged generically from participants concerns and experiences.	Physician-directed	No	No effect
Holt & Del Mar [88]	To reduce psychological distress	Mailing general practitioners (GPs) a letter providing feedback on their baseline psychological score along with a self-help sheet entitled ‘Self, Relationships and Work’.	Physician-directed	No	No effect
Linzer, et al., [52]	To improve clinicians’ office and work-life to enhance satisfaction, retention and burnout	Choosing a variety of method to improve communication and change clinicians’ workflow including: -Adding discussion about work-life issues and personal challenges, or difficult patient care management in the standing monthly meeting agenda; off-loading nonessential tasks to non-physician staff; removing bottlenecks to care in patient rooms regarding medication reconciliation, vaccinations, and data entry; reducing time pressure by increasing visit time by five minutes; instituting a new prescription line to free up RN staff; having clerks instead of clinicians track forms and sending faxes; presenting office and work-life (OWL) data as a platform to discuss issues within the department.	System-directed	No	Favorable outcome for the intervention group
Lucas, et al., [53]	To reduce burnout severity and emotional exhaustion	Assigning random sequences of 2- and 4-week in-patient rotations	System-directed	No	Favorable outcome for the intervention group
Margalit, et al., [63]	To reduce burnout, work related strain and mental workload	Participating in didactic and interactive program including reading assignments, lectures and discussions, in addition to role-playing exercises and one-to-one counseling by a facilitator	Physician-directed	Yes (Biopsychological approach)	Negative effect on burnout
Martins, et al., [65]	To control burnout	Participating in a two 2.5 h workshop facilitated by health professionals informing participants of repercussions of burnout, risk detection and identification, coping strategies and tools (self-care behaviors) 2-month self-care workshops (brief intervention)	Physician-directed	No	No effect
Milstein, et al., [66]	To reduces symptoms characteristic of burnout	Receiving training in the use of a self-administered psychotherapeutic tool to reduce symptoms attributable to burnout during a three-month period.	Physician- directed	No	No effect
Montero-Marin, et al., [58]	To enhance positive affect/wellbeing and reduce negative affect	One face-to face meetings (4 h) and eight brief blended unsupported web-based mindfulness practices (two weekly sessions over 4 weeks)	Physician-directed	No	Favorable outcome for the intervention group
Parshuram, et al., [55]	To improve resident wellbeing	Assigning random sequences of 12- or 16-h rotations/schedules in ICU	System-directed	No	No effect
Ripp, et al., [51]	To mitigate burnout	Decreasing the duty hour hours restriction and receiving one day off every week	System-directed	No	No effect
Shea, et al., [54]	To reduce end-of-rotation fatigue, emotional exhaustion, depersonalization, burnout	Assignment Using random sequences for call- rotations to protect sleep period during overnight call rotations	System- directed	No	No effect
Verweij, et al., [59]	To enhance work engagement, mindfulness skill, and empathy	Participating in mindfulness-based stress reduction,	Physician-directed	No	Favorable outcome for the intervention group
Weight, et al., [64]	To improve quality of life and reduce burnout	Providing team-based incentivized 12-week free access to the workplace exercise facility	System-directed	No	Favorable outcome for the intervention group
West, et al., [60]	To promote physician well-being, meaning, engagement, empowerment in work, job satisfaction, and burnout	Participating in 19-biweekly group discussion incorporating elements of mindfulness, reflection, shared experience, and small-group learning with protected 1-h paid time every other week	System-directed Physician-directed	No	Favorable outcome for the intervention group

[50, 56, 57, 62, 68, 67, 49, 61, 88, 52, 53, 63, 65, 66, 58, 55, 51, 54, 59, 64, 60]

Our data reflects that reducing burnout from the perspective of positive psychology has gained momentum and validity among international scholars as it has been in the U.S. However, the use of physician-based and system-based intervention to overcome burnout by various studies speaks of continuous disputes around viewing burnout as a physician-related syndrome or work-setting-related phenomenon, supporting other empirical evidence. [69] Additionally, burnout seems to be an issue despite variability in the work ethic and culture in the high-income countries we covered in this review. This brings in mind the significance of this issue in low to middle-income countries with large health services demands. This scarcity in data demands more burnout related data from these. [70].

## Discussion

In this systematic review, we aimed to characterize studies using interventions within the PERMA framework to ameliorate burnout and improve physician well-being. While the interventions in these studies used strategies that resonate with PERMA elements (i.e., to enhancing participant positive emotion, engagement, positive relation, meaning, and accomplishment) the interventions were not guided by any conceptual framework, including the PERMA. Of the 21 studies, only one used a theoretical-based intervention, where bio-psychosocial approach components were added to the intervention. [63] Other studies targeted participant burnout or well-being by exposing them to positive physical, mental, work, and/or social experiences. In the majority of the studies, participants experienced some level of positive perceptions. They were conceptualized as satisfaction with one’s job, finding meaning in one’s job, staying engaged while at work, experiencing work-life balance, less emotional exhaustion, positive attitude, and improving coping and communication skills as a proxy for professional well-being. [37, 71] However, system-directed interventions produced more favorable results compared to physician-directed interventions.

While theoretical models provide valuable guidance in the developing, implementing, evaluating, and success of novel interventions. [72] [73] [74] [75] [76] our review demonstrates that the use of conceptual frameworks in intervention studies implemented in the healthcare setting is limited, as reported before. [73] [77] The Medical Research Council also argues that interventions grounded in theory are more likely to be effective than those that are solely empirical or pragmatic, as theory helps to understand why failures arise and to identify change mechanisms for improvement purposes. [78] [74, 79].

The recently updated definition of professional burnout by the World Health Organization (WHO) considers burnout a work-related syndrome with ICD-11 (*International Classification of Diseases, Eleventh Revisions*) code of QD85.[80] According to WHO, burnout is characterized by; 1) experiencing a state of exhaustion, 2) increased negativism or cynicism toward one’s job, and 3) perceived low self-efficacy and achievement in once profession.[80] Several components of the PERMA conceptual framework address system-directed intervention that values enabling individuals to thrive, engage, commit, and find meaning in their professions at the personal and organizational level, which are a common proxy for professional well-being, and contrary to experiencing burnout. [81–83] More specifically, the values of finding meaning and purpose in work, in promoting engagement, creativity, and commitment to patient care, were evident in our review, [60] and supported by other empirical studies. [84–86].

To further advance positive psychology’s contributions in mitigating physician burnout, there is a need to identify predictive model that can envisage the underlying mechanism that keeps clinicians motivated, engaged, and productive. While there is no generally agreed-upon definition of professional well-being,[87] examining the efficacy of PERMA elements regarding the attainment of clinician well-being within a system-directed approach could fill the existing empirical gap.

This study has several limitations. Our search does not include Web of Science or Scopus, which could have limited our findings. The inclusion of only a subgroup of positive psychology interventions may have excluded pertinent studies or resulted in selection bias. Additionally, we admit that the significant numbers of articles published in languages other than English contribute to the development of this topic, but due to the inability of the research team, we could not access and evaluate them. In addition, due to the heterogeneity of the interventions and their implementation in varying healthcare settings and departments, a meta-analysis was not performed.

## Conclusion

In conclusion, consideration of the utility of PERMA as a multidimensional model of positive psychology to guide interventions to reduce burnout and enhance well-being among physicians is missing in the literature. Nevertheless, the majority of the studies reported some level of positive outcome regarding reducing burnout or improving well-being by using a physician or a system-directed intervention. Albeit, we found more favorable outcomes in system-directed intervention. Our finding highlights the research paucity in incorporating conceptual models in the design and implementation of positive psychology interventions to mitigate physician burnout. Future studies are needed to evaluate how PERMA as a framework can disentangle individual from interpersonal and institutional levels of analysis and impact (e.g., self-care from group activities in the physician-directed domain, and work or material from social conditions in the systems-directed domain).

## Supplementary Information

**Additional file 1:.**

## Data Availability

All data generated or analyzed during this study are included in this published article [and its supplementary information files].
