# Supplementary material for: Contribution of a positive psychology-based conceptual framework in reducing physician burnout and improving well-being: a systematic review
Source: BMC Med Educ. 2021 Nov 25;21:593. doi: 10.1186/s12909-021-03021-y (PMC8620251; doi:10.1186/s12909-021-03021-y)
Supplement: Supplementary file 1 — Additional file 1:. [file 12909_2021_3021_MOESM1_ESM.docx]

**SUPPLEMENTARY TABLES**

**Supplementary Table 1.** Search Strategy

| **Data Source** | **Search Term** |
| --- | --- |
| PUBMED | (“[Allied Health Personnel](https://www-ncbi-nlm-nih-gov.ezproxy.cdrewu.edu/mesh/68000488)” OR “[Community Health Workers](https://www-ncbi-nlm-nih-gov.ezproxy.cdrewu.edu/mesh/68003150)” OR “[Dental Auxiliaries](https://www-ncbi-nlm-nih-gov.ezproxy.cdrewu.edu/mesh/68003727)” OR “[Emergency Medical Technicians](https://www-ncbi-nlm-nih-gov.ezproxy.cdrewu.edu/mesh/68004634)” OR “[Home Health Aides](https://www-ncbi-nlm-nih-gov.ezproxy.cdrewu.edu/mesh/68018576)” OR “[Licensed Practical Nurses](https://www-ncbi-nlm-nih-gov.ezproxy.cdrewu.edu/mesh/68065102)” OR “[Medical Record Administrators](https://www-ncbi-nlm-nih-gov.ezproxy.cdrewu.edu/mesh/68008497)” OR “[Nursing Assistants](https://www-ncbi-nlm-nih-gov.ezproxy.cdrewu.edu/mesh/68009728)” OR “[Operating Room Technicians](https://www-ncbi-nlm-nih-gov.ezproxy.cdrewu.edu/mesh/68009872)” OR “[Pharmacy Technicians](https://www-ncbi-nlm-nih-gov.ezproxy.cdrewu.edu/mesh/68010596)” OR “[Physical Therapist Assistants](https://www-ncbi-nlm-nih-gov.ezproxy.cdrewu.edu/mesh/68063372)” OR “[Physician Assistants](https://www-ncbi-nlm-nih-gov.ezproxy.cdrewu.edu/mesh/68010823)” OR “[Anatomists](https://www-ncbi-nlm-nih-gov.ezproxy.cdrewu.edu/mesh/68054814)” OR “[Anesthetists](https://www-ncbi-nlm-nih-gov.ezproxy.cdrewu.edu/mesh/2016596)” OR “[Anesthesiologists](https://www-ncbi-nlm-nih-gov.ezproxy.cdrewu.edu/mesh/2016599)” OR “[Nurse Anesthetists](https://www-ncbi-nlm-nih-gov.ezproxy.cdrewu.edu/mesh/68009719)” OR “[Audiologists](https://www-ncbi-nlm-nih-gov.ezproxy.cdrewu.edu/mesh/2016586)” OR “[Caregivers](https://www-ncbi-nlm-nih-gov.ezproxy.cdrewu.edu/mesh/68017028)” OR “[Case Managers](https://www-ncbi-nlm-nih-gov.ezproxy.cdrewu.edu/mesh/2016577)” OR “[Coroners” OR “Medical Examiners](https://www-ncbi-nlm-nih-gov.ezproxy.cdrewu.edu/mesh/68003334)” OR “[Dental Staff](https://www-ncbi-nlm-nih-gov.ezproxy.cdrewu.edu/mesh/68003797)” OR “[Dentists](https://www-ncbi-nlm-nih-gov.ezproxy.cdrewu.edu/mesh/68003815)” OR “[Endodontists](https://www-ncbi-nlm-nih-gov.ezproxy.cdrewu.edu/mesh/2016647)” OR “[Oral OR Maxillofacial Surgeons](https://www-ncbi-nlm-nih-gov.ezproxy.cdrewu.edu/mesh/2009662)” OR “[Orthodontists](https://www-ncbi-nlm-nih-gov.ezproxy.cdrewu.edu/mesh/2009661)” OR “[Doulas](https://www-ncbi-nlm-nih-gov.ezproxy.cdrewu.edu/mesh/68059037)” OR “[Emergency Medical Dispatcher](https://www-ncbi-nlm-nih-gov.ezproxy.cdrewu.edu/mesh/2016554)” OR “[Epidemiologists](https://www-ncbi-nlm-nih-gov.ezproxy.cdrewu.edu/mesh/2016626)” OR “[Faculty, Dental](https://www-ncbi-nlm-nih-gov.ezproxy.cdrewu.edu/mesh/68005179)” OR “[Faculty, Medical](https://www-ncbi-nlm-nih-gov.ezproxy.cdrewu.edu/mesh/68005180)” OR “[Faculty, Nursing](https://www-ncbi-nlm-nih-gov.ezproxy.cdrewu.edu/mesh/68005181)” OR “[Health Educators](https://www-ncbi-nlm-nih-gov.ezproxy.cdrewu.edu/mesh/68040441)” OR “[Health Facility Administrators](https://www-ncbi-nlm-nih-gov.ezproxy.cdrewu.edu/mesh/68006270)” OR “[Hospital Administrators](https://www-ncbi-nlm-nih-gov.ezproxy.cdrewu.edu/mesh/68006740)” OR “[Infection Control Practitioners](https://www-ncbi-nlm-nih-gov.ezproxy.cdrewu.edu/mesh/68016357)” OR “[Medical Chaperones](https://www-ncbi-nlm-nih-gov.ezproxy.cdrewu.edu/mesh/68061348)” OR “[Medical Laboratory Personnel](https://www-ncbi-nlm-nih-gov.ezproxy.cdrewu.edu/mesh/68017056)” OR “[Medical Staff](https://www-ncbi-nlm-nih-gov.ezproxy.cdrewu.edu/mesh/68008503)” OR “[Medical Staff, Hospital](https://www-ncbi-nlm-nih-gov.ezproxy.cdrewu.edu/mesh/68008505)” OR “[Nurses](https://www-ncbi-nlm-nih-gov.ezproxy.cdrewu.edu/mesh/68009726)” OR “[Nurse Administrators](https://www-ncbi-nlm-nih-gov.ezproxy.cdrewu.edu/mesh/68009718)” OR “[Nurse Practitioners](https://www-ncbi-nlm-nih-gov.ezproxy.cdrewu.edu/mesh/68009722)” OR “[Nurse Specialists](https://www-ncbi-nlm-nih-gov.ezproxy.cdrewu.edu/mesh/2016640)” OR “[Nurses, Community Health](https://www-ncbi-nlm-nih-gov.ezproxy.cdrewu.edu/mesh/68064691)” OR “[Nurses, International](https://www-ncbi-nlm-nih-gov.ezproxy.cdrewu.edu/mesh/68064700)” OR “[Nurses, Male](https://www-ncbi-nlm-nih-gov.ezproxy.cdrewu.edu/mesh/68009727)” OR “[Nurses, Public Health](https://www-ncbi-nlm-nih-gov.ezproxy.cdrewu.edu/mesh/68064689)” OR “[Nursing Staff](https://www-ncbi-nlm-nih-gov.ezproxy.cdrewu.edu/mesh/68009740)” OR “[Nursing Staff, Hospital](https://www-ncbi-nlm-nih-gov.ezproxy.cdrewu.edu/mesh/68009741)” OR “[Nutritionists](https://www-ncbi-nlm-nih-gov.ezproxy.cdrewu.edu/mesh/68064686)” OR “[Occupational Therapists](https://www-ncbi-nlm-nih-gov.ezproxy.cdrewu.edu/mesh/2016604)” OR “[Optometrists](https://www-ncbi-nlm-nih-gov.ezproxy.cdrewu.edu/mesh/2016630)” OR “[Personnel, Hospital](https://www-ncbi-nlm-nih-gov.ezproxy.cdrewu.edu/mesh/68010564)” OR “[Dental Staff, Hospital](https://www-ncbi-nlm-nih-gov.ezproxy.cdrewu.edu/mesh/68003798)” OR “[Hospital Administrators](https://www-ncbi-nlm-nih-gov.ezproxy.cdrewu.edu/mesh/68006740)” OR “[Hospital Volunteers](https://www-ncbi-nlm-nih-gov.ezproxy.cdrewu.edu/mesh/68006758)” OR “[Medical Staff, Hospital](https://www-ncbi-nlm-nih-gov.ezproxy.cdrewu.edu/mesh/68008505)” OR “[Nursing Staff, Hospital](https://www-ncbi-nlm-nih-gov.ezproxy.cdrewu.edu/mesh/68009741)” OR “[Pharmacists](https://www-ncbi-nlm-nih-gov.ezproxy.cdrewu.edu/mesh/68010595)” OR “[Physical Therapists](https://www-ncbi-nlm-nih-gov.ezproxy.cdrewu.edu/mesh/68059825)” OR “[Physician Executives](https://www-ncbi-nlm-nih-gov.ezproxy.cdrewu.edu/mesh/68008486)” OR “[Physicians](https://www-ncbi-nlm-nih-gov.ezproxy.cdrewu.edu/mesh/68010820)” OR “[Allergists](https://www-ncbi-nlm-nih-gov.ezproxy.cdrewu.edu/mesh/2016652)” OR “[Anesthesiologists](https://www-ncbi-nlm-nih-gov.ezproxy.cdrewu.edu/mesh/2016599)” OR “[Cardiologists](https://www-ncbi-nlm-nih-gov.ezproxy.cdrewu.edu/mesh/2016605)” OR “[Dermatologists](https://www-ncbi-nlm-nih-gov.ezproxy.cdrewu.edu/mesh/2016602)” OR “[Endocrinologists](https://www-ncbi-nlm-nih-gov.ezproxy.cdrewu.edu/mesh/2016606)” OR “[Foreign Medical Graduates](https://www-ncbi-nlm-nih-gov.ezproxy.cdrewu.edu/mesh/68005550)” OR “[Gastroenterologists](https://www-ncbi-nlm-nih-gov.ezproxy.cdrewu.edu/mesh/2016611)” OR “[General Practitioners](https://www-ncbi-nlm-nih-gov.ezproxy.cdrewu.edu/mesh/68058005)” OR “[Geriatricians](https://www-ncbi-nlm-nih-gov.ezproxy.cdrewu.edu/mesh/2016648)” OR “[Hospitalists](https://www-ncbi-nlm-nih-gov.ezproxy.cdrewu.edu/mesh/68020445)” OR “[Nephrologists](https://www-ncbi-nlm-nih-gov.ezproxy.cdrewu.edu/mesh/2016613)” OR “[Neurologists](https://www-ncbi-nlm-nih-gov.ezproxy.cdrewu.edu/mesh/2016621)” OR “[Occupational Health Physicians](https://www-ncbi-nlm-nih-gov.ezproxy.cdrewu.edu/mesh/68054538)” OR “[Oncologists](https://www-ncbi-nlm-nih-gov.ezproxy.cdrewu.edu/mesh/2016612)” OR “[Ophthalmologists](https://www-ncbi-nlm-nih-gov.ezproxy.cdrewu.edu/mesh/2016631)” OR “[Osteopathic Physicians](https://www-ncbi-nlm-nih-gov.ezproxy.cdrewu.edu/mesh/68055360)” OR “[Otolaryngologists](https://www-ncbi-nlm-nih-gov.ezproxy.cdrewu.edu/mesh/2016649)” OR “[Pathologists](https://www-ncbi-nlm-nih-gov.ezproxy.cdrewu.edu/mesh/2016622)” OR “[Pediatricians](https://www-ncbi-nlm-nih-gov.ezproxy.cdrewu.edu/mesh/2016623)” OR “[Physiatrists](https://www-ncbi-nlm-nih-gov.ezproxy.cdrewu.edu/mesh/2016625)” OR “[Physicians, Family](https://www-ncbi-nlm-nih-gov.ezproxy.cdrewu.edu/mesh/68010821)” OR “[Physicians, Primary Care](https://www-ncbi-nlm-nih-gov.ezproxy.cdrewu.edu/mesh/68058007)” OR “[Physicians, Women](https://www-ncbi-nlm-nih-gov.ezproxy.cdrewu.edu/mesh/68010822)” OR “[Pulmonologists](https://www-ncbi-nlm-nih-gov.ezproxy.cdrewu.edu/mesh/2016615)” OR “[Radiologists](https://www-ncbi-nlm-nih-gov.ezproxy.cdrewu.edu/mesh/2016633)” OR “[Rheumatologists](https://www-ncbi-nlm-nih-gov.ezproxy.cdrewu.edu/mesh/2016620)” OR “[Surgeons](https://www-ncbi-nlm-nih-gov.ezproxy.cdrewu.edu/mesh/68066231)” OR “[Urologists](https://www-ncbi-nlm-nih-gov.ezproxy.cdrewu.edu/mesh/2016634)”) AND (“PERMA” OR “Positive psychology” OR “well-being theory”) |
| CINAHL | (“[Allied Health Personnel](https://www-ncbi-nlm-nih-gov.ezproxy.cdrewu.edu/mesh/68000488)” OR “[Community Health Workers](https://www-ncbi-nlm-nih-gov.ezproxy.cdrewu.edu/mesh/68003150)” OR “[Dental Auxiliaries](https://www-ncbi-nlm-nih-gov.ezproxy.cdrewu.edu/mesh/68003727)” OR “[Emergency Medical Technicians](https://www-ncbi-nlm-nih-gov.ezproxy.cdrewu.edu/mesh/68004634)” OR “[Home Health Aides](https://www-ncbi-nlm-nih-gov.ezproxy.cdrewu.edu/mesh/68018576)” OR “[Licensed Practical Nurses](https://www-ncbi-nlm-nih-gov.ezproxy.cdrewu.edu/mesh/68065102)” OR “[Medical Record Administrators](https://www-ncbi-nlm-nih-gov.ezproxy.cdrewu.edu/mesh/68008497)” OR “[Nursing Assistants](https://www-ncbi-nlm-nih-gov.ezproxy.cdrewu.edu/mesh/68009728)” OR “[Operating Room Technicians](https://www-ncbi-nlm-nih-gov.ezproxy.cdrewu.edu/mesh/68009872)” OR “[Pharmacy Technicians](https://www-ncbi-nlm-nih-gov.ezproxy.cdrewu.edu/mesh/68010596)” OR “[Physical Therapist Assistants](https://www-ncbi-nlm-nih-gov.ezproxy.cdrewu.edu/mesh/68063372)” OR “[Physician Assistants](https://www-ncbi-nlm-nih-gov.ezproxy.cdrewu.edu/mesh/68010823)” OR “[Anatomists](https://www-ncbi-nlm-nih-gov.ezproxy.cdrewu.edu/mesh/68054814)” OR “[Anesthetists](https://www-ncbi-nlm-nih-gov.ezproxy.cdrewu.edu/mesh/2016596)” OR “[Anesthesiologists](https://www-ncbi-nlm-nih-gov.ezproxy.cdrewu.edu/mesh/2016599)” OR “[Nurse Anesthetists](https://www-ncbi-nlm-nih-gov.ezproxy.cdrewu.edu/mesh/68009719)” OR “[Audiologists](https://www-ncbi-nlm-nih-gov.ezproxy.cdrewu.edu/mesh/2016586)” OR “[Caregivers](https://www-ncbi-nlm-nih-gov.ezproxy.cdrewu.edu/mesh/68017028)” OR “[Case Managers](https://www-ncbi-nlm-nih-gov.ezproxy.cdrewu.edu/mesh/2016577)” OR “[Coroners” OR “Medical Examiners](https://www-ncbi-nlm-nih-gov.ezproxy.cdrewu.edu/mesh/68003334)” OR “[Dental Staff](https://www-ncbi-nlm-nih-gov.ezproxy.cdrewu.edu/mesh/68003797)” OR “[Dentists](https://www-ncbi-nlm-nih-gov.ezproxy.cdrewu.edu/mesh/68003815)” OR “[Endodontists](https://www-ncbi-nlm-nih-gov.ezproxy.cdrewu.edu/mesh/2016647)” OR “[Oral OR Maxillofacial Surgeons](https://www-ncbi-nlm-nih-gov.ezproxy.cdrewu.edu/mesh/2009662)” OR “[Orthodontists](https://www-ncbi-nlm-nih-gov.ezproxy.cdrewu.edu/mesh/2009661)” OR “[Doulas](https://www-ncbi-nlm-nih-gov.ezproxy.cdrewu.edu/mesh/68059037)” OR “[Emergency Medical Dispatcher](https://www-ncbi-nlm-nih-gov.ezproxy.cdrewu.edu/mesh/2016554)” OR “[Epidemiologists](https://www-ncbi-nlm-nih-gov.ezproxy.cdrewu.edu/mesh/2016626)” OR “[Faculty, Dental](https://www-ncbi-nlm-nih-gov.ezproxy.cdrewu.edu/mesh/68005179)” OR “[Faculty, Medical](https://www-ncbi-nlm-nih-gov.ezproxy.cdrewu.edu/mesh/68005180)” OR “[Faculty, Nursing](https://www-ncbi-nlm-nih-gov.ezproxy.cdrewu.edu/mesh/68005181)” OR “[Health Educators](https://www-ncbi-nlm-nih-gov.ezproxy.cdrewu.edu/mesh/68040441)” OR “[Health Facility Administrators](https://www-ncbi-nlm-nih-gov.ezproxy.cdrewu.edu/mesh/68006270)” OR “[Hospital Administrators](https://www-ncbi-nlm-nih-gov.ezproxy.cdrewu.edu/mesh/68006740)” OR “[Infection Control Practitioners](https://www-ncbi-nlm-nih-gov.ezproxy.cdrewu.edu/mesh/68016357)” OR “[Medical Chaperones](https://www-ncbi-nlm-nih-gov.ezproxy.cdrewu.edu/mesh/68061348)” OR “[Medical Laboratory Personnel](https://www-ncbi-nlm-nih-gov.ezproxy.cdrewu.edu/mesh/68017056)” OR “[Medical Staff](https://www-ncbi-nlm-nih-gov.ezproxy.cdrewu.edu/mesh/68008503)” OR “[Medical Staff, Hospital](https://www-ncbi-nlm-nih-gov.ezproxy.cdrewu.edu/mesh/68008505)” OR “[Nurses](https://www-ncbi-nlm-nih-gov.ezproxy.cdrewu.edu/mesh/68009726)” OR “[Nurse Administrators](https://www-ncbi-nlm-nih-gov.ezproxy.cdrewu.edu/mesh/68009718)” OR “[Nurse Practitioners](https://www-ncbi-nlm-nih-gov.ezproxy.cdrewu.edu/mesh/68009722)” OR “[Nurse Specialists](https://www-ncbi-nlm-nih-gov.ezproxy.cdrewu.edu/mesh/2016640)” OR “[Nurses, Community Health](https://www-ncbi-nlm-nih-gov.ezproxy.cdrewu.edu/mesh/68064691)” OR “[Nurses, International](https://www-ncbi-nlm-nih-gov.ezproxy.cdrewu.edu/mesh/68064700)” OR “[Nurses, Male](https://www-ncbi-nlm-nih-gov.ezproxy.cdrewu.edu/mesh/68009727)” OR “[Nurses, Public Health](https://www-ncbi-nlm-nih-gov.ezproxy.cdrewu.edu/mesh/68064689)” OR “[Nursing Staff](https://www-ncbi-nlm-nih-gov.ezproxy.cdrewu.edu/mesh/68009740)” OR “[Nursing Staff, Hospital](https://www-ncbi-nlm-nih-gov.ezproxy.cdrewu.edu/mesh/68009741)” OR “[Nutritionists](https://www-ncbi-nlm-nih-gov.ezproxy.cdrewu.edu/mesh/68064686)” OR “[Occupational Therapists](https://www-ncbi-nlm-nih-gov.ezproxy.cdrewu.edu/mesh/2016604)” OR “[Optometrists](https://www-ncbi-nlm-nih-gov.ezproxy.cdrewu.edu/mesh/2016630)” OR “[Personnel, Hospital](https://www-ncbi-nlm-nih-gov.ezproxy.cdrewu.edu/mesh/68010564)” OR “[Dental Staff, Hospital](https://www-ncbi-nlm-nih-gov.ezproxy.cdrewu.edu/mesh/68003798)” OR “[Hospital Administrators](https://www-ncbi-nlm-nih-gov.ezproxy.cdrewu.edu/mesh/68006740)” OR “[Hospital Volunteers](https://www-ncbi-nlm-nih-gov.ezproxy.cdrewu.edu/mesh/68006758)” OR “[Medical Staff, Hospital](https://www-ncbi-nlm-nih-gov.ezproxy.cdrewu.edu/mesh/68008505)” OR “[Nursing Staff, Hospital](https://www-ncbi-nlm-nih-gov.ezproxy.cdrewu.edu/mesh/68009741)” OR “[Pharmacists](https://www-ncbi-nlm-nih-gov.ezproxy.cdrewu.edu/mesh/68010595)” OR “[Physical Therapists](https://www-ncbi-nlm-nih-gov.ezproxy.cdrewu.edu/mesh/68059825)” OR “[Physician Executives](https://www-ncbi-nlm-nih-gov.ezproxy.cdrewu.edu/mesh/68008486)” OR “[Physicians](https://www-ncbi-nlm-nih-gov.ezproxy.cdrewu.edu/mesh/68010820)” OR “[Allergists](https://www-ncbi-nlm-nih-gov.ezproxy.cdrewu.edu/mesh/2016652)” OR “[Anesthesiologists](https://www-ncbi-nlm-nih-gov.ezproxy.cdrewu.edu/mesh/2016599)” OR “[Cardiologists](https://www-ncbi-nlm-nih-gov.ezproxy.cdrewu.edu/mesh/2016605)” OR “[Dermatologists](https://www-ncbi-nlm-nih-gov.ezproxy.cdrewu.edu/mesh/2016602)” OR “[Endocrinologists](https://www-ncbi-nlm-nih-gov.ezproxy.cdrewu.edu/mesh/2016606)” OR “[Foreign Medical Graduates](https://www-ncbi-nlm-nih-gov.ezproxy.cdrewu.edu/mesh/68005550)” OR “[Gastroenterologists](https://www-ncbi-nlm-nih-gov.ezproxy.cdrewu.edu/mesh/2016611)” OR “[General Practitioners](https://www-ncbi-nlm-nih-gov.ezproxy.cdrewu.edu/mesh/68058005)” OR “[Geriatricians](https://www-ncbi-nlm-nih-gov.ezproxy.cdrewu.edu/mesh/2016648)” OR “[Hospitalists](https://www-ncbi-nlm-nih-gov.ezproxy.cdrewu.edu/mesh/68020445)” OR “[Nephrologists](https://www-ncbi-nlm-nih-gov.ezproxy.cdrewu.edu/mesh/2016613)” OR “[Neurologists](https://www-ncbi-nlm-nih-gov.ezproxy.cdrewu.edu/mesh/2016621)” OR “[Occupational Health Physicians](https://www-ncbi-nlm-nih-gov.ezproxy.cdrewu.edu/mesh/68054538)” OR “[Oncologists](https://www-ncbi-nlm-nih-gov.ezproxy.cdrewu.edu/mesh/2016612)” OR “[Ophthalmologists](https://www-ncbi-nlm-nih-gov.ezproxy.cdrewu.edu/mesh/2016631)” OR “[Osteopathic Physicians](https://www-ncbi-nlm-nih-gov.ezproxy.cdrewu.edu/mesh/68055360)” OR “[Otolaryngologists](https://www-ncbi-nlm-nih-gov.ezproxy.cdrewu.edu/mesh/2016649)” OR “[Pathologists](https://www-ncbi-nlm-nih-gov.ezproxy.cdrewu.edu/mesh/2016622)” OR “[Pediatricians](https://www-ncbi-nlm-nih-gov.ezproxy.cdrewu.edu/mesh/2016623)” OR “[Physiatrists](https://www-ncbi-nlm-nih-gov.ezproxy.cdrewu.edu/mesh/2016625)” OR “[Physicians, Family](https://www-ncbi-nlm-nih-gov.ezproxy.cdrewu.edu/mesh/68010821)” OR “[Physicians, Primary Care](https://www-ncbi-nlm-nih-gov.ezproxy.cdrewu.edu/mesh/68058007)” OR “[Physicians, Women](https://www-ncbi-nlm-nih-gov.ezproxy.cdrewu.edu/mesh/68010822)” OR “[Pulmonologists](https://www-ncbi-nlm-nih-gov.ezproxy.cdrewu.edu/mesh/2016615)” OR “[Radiologists](https://www-ncbi-nlm-nih-gov.ezproxy.cdrewu.edu/mesh/2016633)” OR “[Rheumatologists](https://www-ncbi-nlm-nih-gov.ezproxy.cdrewu.edu/mesh/2016620)” OR “[Surgeons](https://www-ncbi-nlm-nih-gov.ezproxy.cdrewu.edu/mesh/68066231)” OR “[Urologists](https://www-ncbi-nlm-nih-gov.ezproxy.cdrewu.edu/mesh/2016634)”) AND (“PERMA” OR “Positive psychology” OR “well-being theory”) |
| OVID PsycINFO | medical personnel/ or health personnel/ or dentists/ or military medical personnel/ or nurses/ or optometrists/ or pharmacists/ or physical therapists/ or physicians/ or psychiatric hospital staff/ or mental health personnel/ or occupational therapists/ or allied health personnel/ or therapists/ or mental health personnel/ or psychiatric hospital staff/ or physicians/ or family physicians/ or general practitioners/ or gynecologists/ or internists/ or neurologists/ or obstetricians/ or pathologists/ or pediatricians/ or psychiatrists/ or surgeons/ and exp Positive Psychology/ or exp Well Being/ |

**Supplementary Table 2.** Grading of Study Design Quality

| **Grade** | **Design** |
| --- | --- |
| AA | Systematic review or meta-analysis of RCTs |
| A | Systematic review or meta-analysis of non-RCTs |
|  | RCT or cluster RCT |
| B | Systematic review or meta-analysis of controlled studies without a pretest or uncontrolled study with a pretest |
|  | Non-RCT |
|  | Controlled before-&-after study |
|  | Retrospective or prospective cohort study |
|  | Interrupted time series |
|  | Case-control study |
| C | Systematic review or meta-analysis of cross-sectional studies |
|  | Uncontrolled before-&-after study |
| D | Cross-sectional study, models |
| E | Case studies, case reports, narrative reviews |

*RCT = randomized clinical trial
